# Kirschner wire vs screw osteosynthesis of lateral condyle fractures in paediatric patients: a systematic review

**DOI:** 10.1007/s12306-024-00859-5

**Published:** 2024-08-08

**Authors:** D. L. Mostofi Zadeh Haghighi, J. Xu, R. Campbell, T. R. Moopanar

**Affiliations:** 1 Sydney Medical School, The University of Sydney, Camperdown, NSW 2050 Australia; 2 Department of Orthopaedic and Trauma Surgery, Royal North Shore Hospital, St Leonards, NSW 2065 Australia

**Keywords:** Kirschner wire, Cannulated screw, Lateral condyle fracture, Paediatric, Humerus

## Abstract

This systematic review compares Kirschner wires versus a single cannulated screw for the treatment of lateral humeral condyle fractures in children. The purpose of this review is to review the current literature on fixation of lateral condyle fractures of the humerus, and to ascertain whether there is a difference in clinical outcomes of these fractures when fixated with K-wires vs screws. This systematic review of the literature comparing surgical management of paediatric (0–17 years of age) lateral condyle fractures with K-wire versus screw fixation was performed using the Preferred Reporting Items for Systematic Reviews and Meta-Analyses (PRISMA) guidelines. Electronic searches of three databases from inception to March 2022 yielded 17 studies which satisfied inclusion criteria, comprising 1,272 patients with a median age of 8.5 years. Eight hundred and fifty-five (67.2%) patients underwent K-wire fixation and 417 (32.8%) underwent screw fixation. Results were divided into comparative and single-arm studies. The median follow-up time was 23.3 months (range 3 months–22 years). A lateral prominence was observed in 114 (13.3%) patients with K-wires and 41 (9.8%) patients with a cannulated screw. An infection developed in 52 (6.1%) patients with K-wires, while only five (1.2%) patients with a screw developed an infection. A carrying angle deformity occurred in 61 (7.1%) patients with K-wires and seven (1.7%) patients with a screw. K-wires and cannulated screws are effective and safe methods of fixation for lateral humeral condyle fractures in children. K-wire fixation may have a greater incidence of infection but allows for safe non-operative removal and versatility with fractures of greater comminution, while screw fixation necessitates a second operation for removal following union.

*Level of Evidence* III Systematic review.

## Introduction

Lateral condyle fractures are the second most common type of paediatric elbow fractures [1]. Compared to other paediatric elbow fractures, there is a high risk of malunion, non-union, and angular deformities [2]. The current literature suggests that displaced fractures (> 2 mm) may benefit from operative management, while undisplaced or minimally displaced fractures (< 2 mm) can achieve a satisfactory outcome with long arm cast immobilization and close follow-up [2, 3]. Operative management may be achieved with careful closed reduction techniques and percutaneous fixation, with consideration of an intraoperative arthrogram to aid in evaluation of displacement [4]. However, displaced lateral condyle fractures often require open reduction techniques with internal fixation using Kirschner wires (K-wires) or cannulated screws (CS) [5, 6]. K-wire osteosynthesis commonly involves 2 to 3 percutaneous pins, whereas cannulated screw fixation is sometimes preferred for large fragments, or in the event of non-union [7, 8]. Regardless of fixation method, utmost care must be taken to avoid significant posterior dissection, as this is the predominate source of blood supply [9].

There remains no consensus on the ideal fixation method in the surgical management of lateral condyle fractures [6]. K-wires carry advantages in their ability to be placed with more ease and removal without undergoing a second operation [3]. It is particularly favoured in younger patients, with smaller fracture fragments or cases of comminution. However, it has been associated with wire site infection, particularly when left proud, and loss of fixation [1, 10]. Screw fixation, on the other hand, provides greater compression and more rigid fixation, thus being attributed with lower rates of non-union [11]. However there is concern that screw fixation can increase the risk of developing avascular necrosis (AVN) and physeal arrest, leading to angular deformity [1, 12].

The aim of this study is to review current literature to compare K-wire fixation with screw osteosynthesis for fixation of lateral condyle fractures in children, treated via open or percutaneous techniques. We aim to evaluate their respective outcomes and complications, and to gain a better understanding of the clinical implications when tailoring the choice of fixation to the individual patient.

## Methods

### Search strategy

**Fig. 1:**
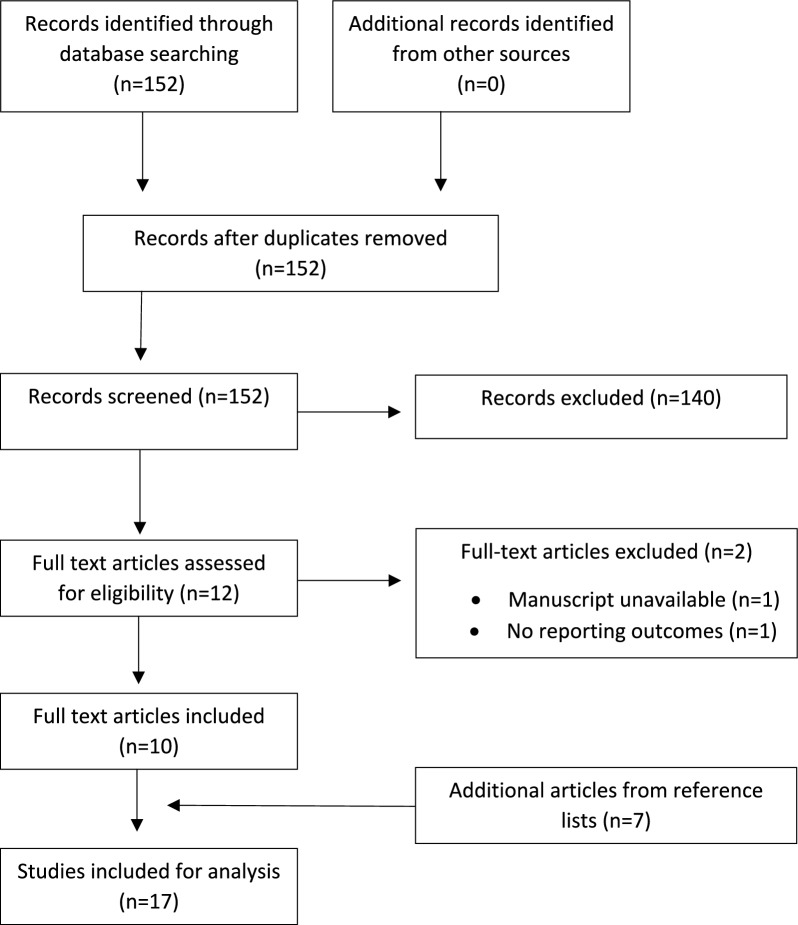
PRISMA flowchart demonstrating the methodology of the literature search

The study was conducted according to the Preferred Reporting Items for Systematic Reviews and Meta-analyses guidelines (PRSIMA) (Fig. 1). Electronic databases (Ovid Medline, PubMed, and Cochrane Central Register of Controlled Trials) were searched to include all relevant studies up to March 2022. Using Boolean operators, the searches combined terms for K-wire, cannulated screw, lateral condyle fracture. The reference lists of included studies were evaluated for potentially relevant studies to be hard-searched and included.

### Study selection

Inclusion criteria was specified at study conception and agreed upon by all reviewers. Inclusion criteria were paediatric patients (17 years or younger) receiving K-wire and/or screw osteosynthesis following traumatic fracture of the lateral condyle of the humerus. This included patients treated via closed or open reduction techniques. Both comparative and single-arm studies were included. When institutions published duplicate studies with accumulating numbers of patients or increased lengths of follow-up, only the most complete reports were included for quantitative assessment at each time interval. The selection of studies was limited to those published in either the English or German language and reported at least one efficacy or safety outcome of interest. Abstracts, case reports, conference presentations, editorials and expert opinions were excluded. Review articles were omitted because of potential publication bias and duplication of results.

### Data extraction

Data were extracted by two independent authors (DM, JX) from the included studies. Any disagreements were resolved by discussion with the senior author (TM). Study characteristics and data on outcomes were extracted. Primary efficacy outcomes included rates of lateral prominence, carrying angle deformities, non-union and superficial infection. Secondary outcomes were all other deformities, delayed unions and causes for revision surgery.

## Results

### Search results

A literature search identified 152 initial results. Of these studies, 17 met our inclusion criteria [1–3, 5–8, 10, 13–21]. Demographic data are summarized in Table 1. Of the 17 included studies, nine were two-arm studies comparing K-wire and screw fixation, seven were single-arm studies assessing K-wire fixation, and one was a single-arm study assessing screw fixation. Complications are summarized in Table 2.

**Table 1:** Patient demographic and clinical data

	K-wire		Screw	1-arm KW	1-arm CS
Patient number	414		321	441	96
Median age (yrs)		8.5		4.7	5.8
Male		475		275	70
Female		258		154	26
Median follow-up (mo)		39.4		16.4	6.5
Right side		176		152	38
Left side		199		233	58
*Fracture classification*					
Milch I	80		11	17	0
Milch II	176		115	101	0
Jakobs II	42		38	108	45
Jakobs III	45		49	130	51
Song III	0		0	11	0
Song IV	0		0	6	0

**Table 2:** Complications

	K-wire n (%)	Screw n (%)	1-arm KW n (%)	1-arm CS n (%)
Patients	414	321	441	96
Non-union	15 (3.6)	0	0	0
Malunion	3 (0.7)	2 (0.6)	0	0
Delayed union	5 (1.2)	3 (0.9)	1 (0.2)	1 (1.0)
Lateral prominence	22 (5.3)	29 (9.0)	92 (20.9)	12 (12.5)
Carrying angle deformities	12 (2.9)	7 (1.7)	49 (11.1)	0
Cubitus varus	7 (1.7)	3 (0.9)	33 (7.5)	0
Cubitus valgus	5 (1.2)	4 (1.3)	6 (1.4)	0
Fishtail deformity infection	4 (1.0)	4 (1.3)	24 (5.4)	0
Superficial	18 (4.3)	3 (0.9)	25 (5.7)	0
Deep	3 (0.7)	2 (0.6)	6 (1.4)	
Revision surgery	7 (1.7)	0	1 (0.2)	0
AVN	3 (0.7)	0	3 (0.7)	0

### Comparative studies

#### Baseline characteristics

A total of nine studies compared K-wire and screw fixation. From these studies, there were 735 patients, of which 414 (56.3%) underwent K-wire fixation and 321 (43.7%) underwent screw fixation. (Table 1) Fractures were classified according to either the Milch or the Jakobs classification (Table 1). Immobilization lasted a median of 9.25 weeks (range: 2.5–16) following K-wire fixation and for a median of 6.5 weeks (range: 1–12) following screw fixation. Hardware removal was based on radiographic evidence of union and occurred a median of 11 weeks (range: 3–19) after K-wire fixation and a median of 18.2 weeks (6–30.4 weeks) after screw fixation.

#### Outcomes

From three studies, 163 (73 KW, 90 CS) patients were evaluated using the criteria from Hardacre et al [22]. In the KW group, 55 (75.3%) patients had excellent outcomes, 15 (20.6%) patients had good outcomes, no patients had fair outcomes, and three (4.1%) patients had poor outcomes. In the CS group, 74 (82.2%) patients had excellent outcomes, 16 (17.8%) patients had good outcomes, and no patients had fair or poor outcomes. The three poor outcomes in the KW group were due to non-union. A ROM deficit compared to their contralateral side was reported in 35 patients. ROM deficit occurred in 5.1% in the KW group (range: 4.8–36.4%, four studies) and in 4.4% in the CS group (range: 4.5–30.8%, four studies). Complications are summarized in Table 2. The overall complication rate was 22.2% in the KW group and 15.6% in the CS group. The primary complication for both groups was the development of a lateral prominence.

### Single-arm K-wire studies

#### Baseline characteristics

From the seven single-arm studies, there were 441 patients included (Table 1). There were 286 males (64.9%) and 155 females (35.1%). The median age of patients at time of injury was 8.25 years (range 1.5–15yrs), and patients were followed up for a median of 6.41 years (range 1wk–12.8yrs). Classification of fractures varied between studies, 373 fractures total were classified according to the Milch, Jakobs or Song classification, and displacement was > 2 mm in all fractures. Post-operative immobilization was achieved using a long arm cast or slab for a median of 4.5 weeks (range 3–6 weeks). Union as confirmed with radiographic evidence at a median of 7.3 weeks (3.6–11 weeks), hardware was removed a median of 16 weeks post-op (range 6–26 weeks).

#### Outcomes

Two studies used the criteria from Dhillon et al [23] and one study used the Flynn criteria [24]. There were 281 patients evaluated using these criteria. One hundred and ninety-nine (70.8%) patients had excellent outcomes, 78 (27.8%) patients had good outcomes, four (1.4%) patients had fair outcomes, and no patients had poor outcomes. Complications are summarized in Table 2. The overall complication rate was 45.6%. The primary complication in this group was the development of a lateral prominence.

### Single-arm screw studies

Only one study evaluating screw fixation alone was included. Demographic data are summarized in Table 1. Fractures were classified via the Jakobs classification [25], with consolidation occurring at a mean of 8.9 weeks (range 3.9–13.9 weeks), and hardware removed at a median of 79.3 weeks (range 6.4–152.2 weeks). Thirty patients (31%) had an impairment of ROM compared to the uninjured side, of which 20 patients had a mean flexion loss of eight degrees and 30 had a mean extension loss of two degrees. Twelve (13%) patients developed a lateral prominence, and one (1.0%) patient had a delayed union.

## Discussion

This study has reviewed literature on studies assessing the clinical outcomes and complications following both K-wire and cannulated screw fixation of lateral condyle fractures in children. We found that clinical outcomes of both fixation modalities were acceptable in the majority of patients. The pooled complication rate in the KW group was 34.3% compared to 15.1% in the CS group. The primary complication for both groups was the development of a lateral prominence. This review has also highlighted the limited quality of evidence on this topic. The heterogeneity of the available data made any meta-analysis of outcomes inappropriate.

K-wires are historically the preferred method of fixation in children with lateral condyle fractures. An advantage of fixation with K-wires instead of screws is the ability for the wires to be removed in clinic, eliminating the need for a second operation [6]. Furthermore, patients may be lost to follow-up. This may lead to deformity if a transphyseal screw is left in situ. Shirley et al. reported that 40 patients in their study had screws left in situ of which 61% did not return to arrange removal although being advised to do so [8]. It is thought that leaving screws in situ increases the risk of impression or distortion of growth across the physis, although the actual risk remains contested [3, 7].

The choice of open versus closed reduction remains controversial [4]. Song et al. state that open reduction may be unnecessary and could be avoided with careful attention to closed reduction manoeuvres in many cases [26]. In another study, Song et al. evaluated closed reduction with percutaneous K-Wire pinning in 63 patients with unstable displaced fractures. Only 13 cases required the surgeons to revert to an open approach when anatomical reduction could not be achieved [27]. The aid of an arthrogram can greatly aid in assessing whether adequate anatomical reduction in these partial articular fractures has been achieved. However, there remains an element of surgeon experience which dictates the effectiveness of closed reduction when compared to open reduction [27, 28]. Great care must be taken to avoid overzealous posterior dissection in open reduction given the predominate posterior blood supply and subsequent risk of AVN. We also advocate for use of a headlamp and/or loupes to enable visual confirmation of anatomic reduction when using an open approach. However, Zhu et al. concluded that there is no significant difference (*P* > 0.05) between closed or open reduction regarding infection, AVN and non-union rates [29].

Screw fixation has the advantage of compression across the fracture site and increased biomechanical stability [3]. This may explain why screws are seldom associated with loss of fixation, making them ideal for fractures with a large fragment. *Stein *et al. observed one case where loss of fixation with a screw occurred. They attributed it to the use of a 3.5-mm screw and did not observe this complication with use of the 4.5-mm screw [3]. *Gilbert *et al. and *Stein *et al. attributed screw fixation to faster rates of consolidation as well as lower instances of infection and open reduction [3, 10]. Further, screw fixation provides compression across the fracture while still allowing the patient to initiate range of motion with the screw still in situ [3].

Development of a lateral prominence was the most common complication in this study. *Hardacre *et al. suggest that lateral overgrowth may be attributed to transient stimulation of the lateral physis [22]. *Hasler and von Laer* reported that when treated with screws under compression, 15% of their patients developed a lateral prominence compared with 50% of their 14 patients treated using screws without compression [14]. *Shirley *et al. analysed the prevalence of lateral prominence following K-wire fixation in current literature and found it ranged from 10 to 86% [8]. This supports our findings, the incidence of lateral prominence when using K-wires ranged from 2.1 to 80%, and with screws ranged from 9.9% to 33.3%. These findings suggest that using screws under compression may reduce the risk of lateral overgrowth compared to the rates observed when using K-wires.

The rate of superficial infections when using exposed K-wires is an important consideration on surgical choice.

Burying K-wires may reduce the incidence of superficial infections; however, these require a second surgery for removal, eliminating the benefit K-wires had, being able to be removed in clinic [10]. In our analysis, deep infections in the KW group ranged from 1.4 to 10%, and in the CS group ranged from 2.4 to 4.5%. We found that revision surgery for wound infections was reported in the KW group ranging from 1.4 to 4.5%, while none were reported in the CS group. This is in line with the findings of Sinha et al. in a recent systematic review published on the topic30. This review considers a broader publication date range and evaluates a greater number of fractures, building upon the previous review.

It is important to recognize the limitations of this study. The studies we included scarcely used outcome criteria, and in the handful of studies that did use outcome criteria, different sets of criteria were used. This made it hard to compare the outcomes of multiple studies. Using standardized criteria would aid in a more uniform presentation of data in the future studies. Range of motion on follow-up was seldom reported. Some studies reported mean ranges of motion for their entire cohort, while other studies reported mean deficits in affected patients or only listed the number of deficits, neglecting to add a ROM value. Mean Baumann’s angles and carrying angles were also rarely reported, while cubitus varus and cubitus valgus deformities were consistently reported; this made it difficult to ascertain whether there were any instances of angle differences which did not constitute a deformity. Indication for surgery also varied from study to study. When reporting complications, most studies reported on the number of complications observed in the study period, which was highly heterogeneous.

## Conclusion

This article outlines that K-wires and cannulated screws are both safe and effective methods of fixation for lateral condyle fractures in children, having low complication rates and excellent clinical outcomes. The low quality of studies, lack of literature and heterogeneity of outcomes limit the conclusions that can be drawn from this review. Further information and a direct comparison of outcomes for both methods would likely be achieved by prospective randomized trials in future.

## Data Availability

Date was sourced from PubMed using prespecified search and inclusion criteria.
