# Correction to: Kirschner wire vs screw osteosynthesis of lateral condyle fractures in paediatric patients: a systematic review

**DOI:** 10.1007/s12306-024-00867-5

**Published:** 2024-10-14

**Authors:** D. L. Mostof Zadeh Haghighi, J. Xu, R. Campbell, T. R. Moopanar

**Affiliations:** 1 Sydney Medical School, The University of Sydney, Camperdown, NSW 2050 Australia; 2 Department of Orthopaedic and Trauma Surgery, Royal North Shore Hospital, St Leonards, NSW 2065 Australia

**Correction to: MUSCULOSKELETAL SURGERY**. 10.1007/s12306-024-00859-5.

In the original version of this article, email addresses for all authors were included. Only the corresponding author email address (mostofidavid1@gmail.com) should be included.

The ORCID ID 0000-0001-7304-1661 for co-author R. Campbell was inadvertently omitted.

The original article has been corrected.

